# Supplementary material for: Critical spin fluctuations across the superconducting dome in La2−xSrxCuO4
Source: Nat Commun. 2026 Mar 28;17:4564. doi: 10.1038/s41467-026-71319-w (PMC13194800; doi:10.1038/s41467-026-71319-w)
Supplement: Supplementary file 1 — Supplementary Information [file 41467_2026_71319_MOESM1_ESM.pdf]

# Critical spin fluctuations across the superconducting dome in $La_{2-x}Sr_xCuO_4$

Jacopo Radaelli,[1] Aavishkar A. Patel,[2,3] Mengze Zhu,[1] Oliver J. Lipscombe,[1]
J. Ross Stewart,[4] Subir Sachdev,[5] and Stephen M. Hayden[1]

[1]*H.H. Wills Physics Laboratory, University of Bristol, Tyndall Avenue, Bristol BS8 1TL, United Kingdom*
[2]*Center for Computational Quantum Physics, Flatiron Institute, 162 5th Avenue, New York, NY 10010, USA*
[3]*International Centre for Theoretical Sciences, Tata Institute of Fundamental Research, Bengaluru 560089, India*
[4]*ISIS Pulsed Neutron and Muon Source, Rutherford Appleton Laboratory, Didcot OX11 0QX, United Kingdom*
[5]*Department of Physics, Harvard University, Cambridge, MA 02138, USA*

## SUPPLEMENTARY METHODS

### Dynamic critical scaling theory

The dynamic susceptibility of systems below their upper critical dimension is expected to obey the following scaling behavior:

$$\chi(q,\omega,T,t) = b^{\gamma/\nu}\chi(bq, b^z\omega, b^zT, b^{1/\nu}t), \tag{S1}$$

where $b$ is an arbitrary scaling parameter, $t$ is a tuning control parameter [$t = 0$ corresponds to the quantum critical point (QCP)], $q$ is momentum relative to ordering wavevector, $\gamma$ and $\nu$ are the usual susceptibility and correlation function exponents. That is $\chi \propto t^{-\gamma}$ and $\xi \propto t^{-\nu}$. We expect the usual scaling relationship $\gamma = (2-\eta)\nu$.

If we are at the QCP, we have $t = 0$, since $b$ is arbitrary, we can choose $b^zT = 1$, $b = T^{-1/z}$.

$$\chi(q,\omega,T) = b^{\gamma/\nu}\chi(bq, b^z\omega, b^zT), \tag{S2}$$

$$= T^{-\frac{\gamma}{\nu z}}\chi(T^{-\frac{1}{z}}q, \frac{\omega}{T}, 1), \tag{S3}$$

$$= T^{-\alpha}\Phi\left(\xi_T q, \frac{\hbar\omega}{k_BT}\right), \tag{S4}$$

where $\alpha = \gamma/\nu z$, $\Phi$ is a complex function and $\xi_T \propto T^{-1/z}$ a thermal length.

*Dynamic scaling at the ordering wavevector and QCP.*

At the ordering wavevector ($q = 0$) we have

$$\chi''(q=0,\omega,T) = T^{-\frac{\gamma}{\nu z}}\phi_1\left(\frac{\hbar\omega}{k_BT}\right), \tag{S5}$$

where $\phi_P(x) = \Im[\Phi(0,x)]$ is a scaling function. Equation Eqn. S5 is known as $\omega/T$-scaling.

*$\omega/T$ scaling of $\kappa(\omega)$.*

From Eqns. S4 and S5 we can write

$$\frac{\chi''(q,\omega)}{\chi''(q=0,\omega)} = \phi_3\left(\xi q, \frac{\hbar\omega}{k_BT}\right). \tag{S6}$$

We can associate the second factor in Eqn. S10 with $\phi_3$ in Eqn. S6. In order to interpret $\kappa(\omega)$ as a peak width, we set the factor to be a fraction $\beta$ of the peak susceptibility at $q = \kappa(\omega)$ i.e.

$$\beta = \frac{\chi''(q,\omega)}{\chi''(q=0,\omega)} \tag{S7}$$

$$= \phi_3\left(\xi\kappa, \frac{\hbar\omega}{k_BT}\right). \tag{S8}$$

The implicit Eqn. S8 implies that there is a function $\phi_3(x)$ such that $\xi\kappa = \phi_2(\hbar\omega/k_BT)$ and therefore

$$\kappa(\omega) \propto T^{\frac{1}{z}}\phi_2\left(\frac{\hbar\omega}{k_BT}\right) \tag{S9}$$

Thus $\kappa(\omega)$ also shows $\omega/T$ scaling.

### Phenomenological Model

We parameterize our normal-state data with a phenomenological susceptibility used by Aeppli *et al.* [1] :

$$\chi''(\mathbf{Q},\omega) = \chi''(\mathbf{Q}_\delta,\omega)\frac{1}{[1+\kappa^{-2}(\omega)R(\mathbf{Q})]^2}, \tag{S10}$$

where

$$R(\mathbf{Q}) = \frac{1}{4\delta^2}\{[(H-\tfrac{1}{2})^2 + (K-\tfrac{1}{2})^2 - \delta^2]^2 + 4(H-\tfrac{1}{2})^2(K-\tfrac{1}{2})^2\}. \tag{S11}$$

$R(\mathbf{Q})$ has zeros at the positions $\mathbf{Q}_\delta = (1/2\pm\delta, 1/2)$ and $(1/2\pm\delta, 1/2)$ and this function approximately reproduces the $\mathbf{Q}$ dependence of our data. Near each $\mathbf{Q}_\delta$, $R(\mathbf{Q}_\delta + \mathbf{q}) = |\mathbf{q}|^2$.

We find experimentally that the susceptibility at $\mathbf{Q}_\delta$ has the form:

$$\chi''(\mathbf{Q}_\delta,\omega) = \frac{\chi'(\mathbf{Q}_\delta)\Gamma_\delta\,\omega}{\Gamma_\delta^2 + \omega^2} \tag{S12}$$

The real part of the susceptibility $\chi'(\mathbf{Q}_\delta) \propto T^{-\alpha}$ and the spin relaxation rate varies as $\hbar\Gamma_\delta = ak_BT$. We also posit the form

$$\kappa^2(\omega) = \eta k_B^{-2/z}\left[(\hbar\omega)^{2/z} + (k_BT)^{2/z}\right]. \tag{S13}$$

This has the $\omega/T$ scaling form (cf. Eqn. S9)

$$\kappa(\omega) = \eta T^{1/z}\left[1+\left(\frac{\hbar\omega}{k_BT}\right)^{2/z}\right]. \quad \text{(S14)}$$

Finally, combining Eqn. S12 & S13 and setting $R(\mathbf{Q}) = q^2$ we obtain:

$$\chi''(\mathbf{Q},\omega) \propto \frac{T^{-\alpha}\left(\frac{\hbar\omega}{k_BT}\right)}{a^2+\left(\frac{\hbar\omega}{k_BT}\right)^2} \times \frac{1}{\left(1+\left[1+\left(\frac{\hbar\omega}{k_BT}\right)^{2/z}\right]^{-2}\left(\frac{q}{\eta T^{1/z}}\right)^2\right)^2}. \quad \text{(S15)}$$

Eqn. S15 shows the scaling behaviour described by Eqn. S4.

*$\kappa(\omega)$ fitting*

In order to fit our data we use a generalized form of Eqn. S13:

$$\kappa^2(\omega) = \Delta + a_0^{-2}\left[\left(\frac{\hbar\omega}{E_\kappa}\right)^{2/z} + r\left(\frac{k_BT}{E_\kappa}\right)^{2/z}\right]. \quad \text{(S16)}$$

We fit $\kappa(\omega,T)$ in units of $\text{Å}^{-1}$ to the model in Eqn. S16 fixing $r$ to be 1 as has been found to be approximately true in LSCO x=0.14 [1]. Here $\Delta$ is a tuning parameter which is zero for a QCP, $E_\kappa$ is an energy scale and $a_0$ is the in-plane lattice parameter. This fit gives $z = 1.83 \pm 0.35$, $\Delta = (4 \pm 14) \times 10^{-4}\text{Å}^{-2}$ and $E_\kappa = 68 \pm 17$ meV. Thus $\Delta$ is zero within the resolution of the experiment. For the fitted values of $z$ and $a$, the static correlations decay approximately with width $\kappa(0)^{-1}$.

## Quantum phase transitions in metals with Harris disorder

This section recalls the theoretical model presented in Ref. [2], and describes the computation of its real frequency response functions. There is some overlap of the initial discussion here with the review in Ref. [3].

We focus on quantum fluctuations of the spin density wave (SDW) order parameter across the quantum phase transition from the Fermi liquid [4, 5]. We write the SDW order as $(a = x, y, z)$

$$S_a(\mathbf{r}) = \sum_\ell \phi_{\ell a} e^{i\mathbf{Q}_{\delta\ell}\cdot\mathbf{r}} \quad \text{(S17)}$$

where $S_a$ is the electron spin at position $\mathbf{r}$, $\ell = 1\ldots4$ labels the 4 ordering wavevectors $\mathbf{Q}_{\delta\ell}$ at $(1/2, 1/2\pm\delta)$ and $(1/2\pm\delta, 1/2)$. We are interested in fluctuations of the SDW order parameters $\phi_{\ell a}$ coupled to electrons $c_{\mathbf{k}\sigma}$ with dispersion $\varepsilon(\mathbf{k})$ which has a Fermi surface. Including the effects of spatial disorder, we have a two-dimensional Yukawa-Sachdev-Ye-Kitev model with the imaginary time ($\tau$) Lagrangian [6, 7]

$$\mathcal{L}_{YSYK} = \sum_{\mathbf{k}} c^\dagger_{\mathbf{k}\sigma}\left(\frac{\partial}{\partial\tau}+\varepsilon(\mathbf{k})\right)c_{\mathbf{k}\sigma} + \int d^2\mathbf{r}\,\Big\{\lambda\,[\phi(\mathbf{r})]^2 + [g+g'(\mathbf{r})]\sum_\ell c^\dagger_\sigma(\mathbf{r})\tau^a_{\sigma\sigma'}c_{\sigma'}(\mathbf{r})\,\phi_{\ell a}(\mathbf{r})e^{i\mathbf{Q}_{\delta\ell}\cdot\mathbf{r}} + K\,[\nabla_{\mathbf{r}}\phi(\mathbf{r})]^2 + u\,[\phi(\mathbf{r})]^4 + v(\mathbf{r})c^\dagger_\sigma(\mathbf{r})c_\sigma(\mathbf{r})\Big\}. \quad \text{(S18)}$$

Here $\tau^a$ are the Pauli matrices, $\lambda$ is the parameter employed to tune across the transition, and $g$ is the Yukawa coupling between the fermions and bosons. We have included two sources of spatial randomness. The spatially random potential $v(\mathbf{r})$, with ensemble averages $\overline{v(\mathbf{r})} = 0$, $\overline{v(\mathbf{r})v(\boldsymbol{r}')} = v^2\delta(\mathbf{r}-\boldsymbol{r}')$, acts on the fermion density, and plays a central role in the theory of disorder-induced electron localization [8]. Such fermion localization effects are also present here, but all indications are that such effects are not important for the cuprates. Instead, our focus will be on the more relevant 'Harris disorder', induced by spatial randomness in the position of the quantum critical point. Following Ref. [7], we have represented this by a spatially random Yukawa coupling $g'(\mathbf{r})$ with $\overline{g'(\mathbf{r})} = 0$, $\overline{g'(\mathbf{r})g'(\boldsymbol{r}')} = g'^2\delta(\mathbf{r}-\boldsymbol{r}')$. A more conventional form of Harris disorder is a random tuning parameter $\lambda \to \lambda + \delta\lambda(\mathbf{r})$, but this can be transferred to $g'(\mathbf{r})$ by a $\mathbf{r}$-dependent rescaling of $\phi$. In both forms, Harris disorder leads to boson localization at low temperatures [2, 9], where it must be treated non-perturbatively, as we do below. But in higher temperature regimes, where the bosons do not localize, we can add a large number of flavor labels to the fields so that the $g'(\mathbf{r})$ disorder in (S18) is amenable to self-consistent disorder averaged solution which is exact in a large flavor solution [6, 7, 10].

Patel *et al.* [9] have studied the YSYK model (S18) at $g = 0$ by large scale, high precision quantum Monte Carlo simulations (with no additional flavors), and their results for the imaginary time spin susceptibility appear in Fig. 4E of the main text. We describe below the approach used in Ref. [2] which treats the interactions in a mean-field manner, but accounts for disorder numerically exactly; this approach has the advantage of allowing exact analytic continuation to real frequencies at arbitrary temperatures. The results of Ref. [2] are in general agreement with the exact Monte Carlo results of Ref. [9]: both show an extended quantum Griffiths phase with $\omega/T$ scaling, but the value of $\alpha$ is smaller and more reliable in the Monte Carlo study.

The approach of Ref. [2] is to integrate out the fermions from (S18) (assuming fermionic eigenmodes remain extended), and consider the resulting Landau-

damped Hertz-Millis theory for the boson $\phi$ alone. The spatial disorder in the Yukawa coupling $g'(\mathbf{r})$ will lead to disorder in all couplings in the effective boson theory. For simplicity, we retain only the most relevant 'random mass' disorder in the tuning parameter $\lambda \to \lambda + \delta\lambda(\mathbf{r})$. We also drop the longer-range RKKY couplings between the $\phi$ that will be induced by integrating out the fermions [11]. We discretize the boson theory on a lattice of sites (labeled by $j$), and write the SDW order parameters in terms of a real $\phi$ with a single index $a = 1 \ldots M$ with $M = 12$. In this manner, we obtain the action

$$\begin{aligned}\mathcal{S} =& \mathcal{S}_\phi + \mathcal{S}_{\phi d} \\ \mathcal{S}_\phi =& \int d\tau \Bigg[ \frac{J}{2} \sum_{\langle ij \rangle} (\phi_{ia} - \phi_{ja})^2 + \\ & \sum_j \left\{ \frac{\lambda + \delta\lambda_j}{2} \phi_{ja}^2 + \frac{u}{4M} \left(\phi_{ja}^2\right)^2 \right\} \Bigg] \\ \mathcal{S}_{\phi d} =& \frac{T}{2} \sum_{\Omega} \sum_j \left( \gamma |\Omega| + \Omega^2/c^2 \right) |\phi_{ja}(i\Omega)|^2 , \end{aligned} \tag{S19}$$

where $\Omega$ is a Matsubara frequency at a temperature $T$, $\gamma$ is the Landau damping, and the $\Omega^2/c^2$ term has been inserted as a high frequency cutoff. The random mass disorder satisfies $\overline{\delta\lambda(\mathbf{r})} = 0$, $\overline{\delta\lambda(\mathbf{r})\delta\lambda(\boldsymbol{r}')} = \delta\lambda^2 \delta(\mathbf{r} - \boldsymbol{r}')$. For simplicity, we have assumed a global O($M$) symmetry, but this assumption can be relaxed without significantly modifying the results.

The theory in (S19) has been studied using a strong disorder renormalization group [12–14]. But the same basic results are obtained by the method of Ref. [2] (originally used for a related problem in $d = 1$ in Ref. [15]), which also allows study of the crossover at higher energies to weak disorder, and this will be important for our purposes. Following Refs. [2, 15], we replace $\mathcal{S}_\phi$ by an effective quadratic action, while renormalizing the space dependent mass in a self-consistent manner; this leads to

$$\begin{aligned}\widetilde{\mathcal{S}}_\phi =& \int d\tau \Big[ \frac{J}{2} \sum_{\langle ij \rangle} (\phi_{ia} - \phi_{ja})^2 + \sum_j \frac{\widetilde{\lambda}_j}{2} \phi_{ja}^2 \Big] \\ \widetilde{\lambda}_j =& \lambda + \delta\lambda_j + \frac{u}{M} \sum_a \left\langle \phi_{ja}^2 \right\rangle_{\widetilde{\mathcal{S}}_\phi + \mathcal{S}_{\phi d}} \\ =& \lambda + \delta\lambda_j + uT \sum_{\Omega} \sum_b \frac{\psi_{bi}\psi_{bj}}{\gamma|\Omega| + \Omega^2/c^2 + e_b} , \end{aligned} \tag{S20}$$

where $e_b$ and $\psi_{bj}$ are eigenvalues and eigenfunctions of the $\phi$ quadratic form in $\widetilde{\mathcal{S}}_\phi$, labeled by the index $b = 1 \ldots L^2$ for a $L \times L$ sample. For each disorder realization $\delta s_j$, the values of $\widetilde{\lambda}_j$ are determined by numerically solving (S20), and this also yields results for the eigenvalues $e_b$ and the eigenvectors $\psi_{bj}$. The dynamic spin susceptibility is then computed at a real frequency $\omega$ from

$$\chi_{ij}(\omega) = \sum_b \frac{\psi_{bi}\psi_{bj}}{-i\gamma\omega - \omega^2/c^2 + e_b} , \tag{S21}$$

followed by a Fourier transform from spatial co-ordinates to momenta.

Results from the computation above are presented in Fig. 4 of the main text, and Figs. 1-9 below. The chosen parameter values are $J = 1$, $\gamma = 1$, $c^2 = 10$, $u = 1$ and $\delta\lambda^2 = 0.25$. The quantum critical point at $T = 0$ is at $\lambda_c = -0.4586$ and the quantum Griffiths phase begins at $\lambda_G = -0.4300$ [2]. We used a system size of $160 \times 160$ and averaged over 20 disorder realizations. We can set the energy scale by identifying the highest energy energy spin excitation ($c\sqrt{8J} \approx 9$ with the highest energy spin wave ($\approx 300$ meV [16]). This leads to the estimate $300/9 \approx 33$ meV as the unit of energy for the numerics.

The results for the dynamic spin susceptibility at the ordering wavevector $\mathbf{Q}_\delta$ were shown in Fig. 4 of the main text for $\lambda = \lambda_c$, where we found good $\omega/T$ scaling. Fig. 1 extends these results to $\lambda > \lambda_c$. Now we find $\omega/T$ scaling only for smaller values of $\omega/T$. This restriction of $\omega/T$ scaling to $\omega \lesssim T$ for $\lambda > \lambda_c$ is not at odds with the experimental results in the main text, which also, strictly speaking, establish $\omega/T$ scaling for $\omega \lesssim T$ as the range of $\omega$ values available is not enough to achieve larger values of $\omega/T$ at the larger values of $T$ that are considered.

The value of the exponent $\alpha$ decreases monotonically with increasing distance from the critical point (increasing $\lambda$). In Fig. 2, we show that using a smaller value of $\alpha$ makes little difference to the quality of the fit at small $\omega/T$, which is the region at which scaling is established in the observations.

Similar features are present in the quantum Monte Carlo results [9] discussed in Fig. 4 of the main text, where $\lambda_s$ is analogous to $\lambda_c$. For $\lambda > \lambda_s$ and approaching the beginning of the Griffiths phase at $\lambda = \lambda_G$, only the zeroth Matsubara frequency scales as $\chi(\omega = 0) \sim T^{-\alpha}$ (with a value of $\alpha$ that is smaller than that at $\lambda = \lambda_s$) while the nonzero ones do not, implying that $\omega/T$ scaling is restricted to frequencies $\omega < 2\pi T$.

Fig. 3 shows similar results for the *local* dynamic spin susceptibility

$$\chi''_L(\omega) = \int \frac{d^2\mathbf{q}}{4\pi^2} \chi''(\boldsymbol{q}, \omega) \tag{S22}$$

for $\lambda \geq \lambda_c$.

Now we find that $\omega/T$ scaling holds for all values $\lambda$, an indication that the criticality for $\lambda > \lambda_c$ is from localized spin fluctuations. This is just as expected from a quantum Griffiths critical phase. By scaling, the exponent for the local susceptibility $\alpha + \mu = 2/z$, and this is consistent with our values $\mu = 0.1$ to $0.16$, $\alpha = 0.84$ to $0.59$ and $z = 2.1$ to $2.6$ respectively for the range of $\lambda = -0.4586$ to $-0.4300$ considered.

Fig. 4 extends the analysis of scaling for the local and ordering wavevector susceptibilities at $\lambda = \lambda_c$ to much lower values of $T$, showing a breakdown of $\omega/T$ scaling at low $T$.

This is consistent with results for the boson density of states in Ref. [9], which showed a crossover from the constant density of states associated with marginal Fermi liquid behavior at higher energy, to a regime dominated by Griffiths effects at lower energy. The breakdown of $\omega/T$ scaling here in the dynamic spin susceptibility likely arises from the enhanced boson density of states at smallest energies, and could possibly be captured by experiments at lower temperatures than the ones considered in this work.

Next, we turn to the behavior of the inverse correlation length, $\kappa$, defined by (6) in the main text (also (S10)). Given the parameterization in (S17), the dynamic susceptibility is represented by a sum of contributions in the vicinity of the $\mathbf{Q}_{\delta\ell}$, each of which is discretized on the square lattice. Consequently, it is appropriate to adapt (S10) to

$$\frac{\chi''(\mathbf{q},\omega)}{\chi''(\mathbf{q}=0,\omega)} = \frac{\kappa^4(\omega)}{[\kappa^2(\omega) + 4 - 2\cos(q_x) - 2\cos(q_y)]^2} \,. \quad \text{(S23)}$$

Fig. 5 shows that (S23) provides an excellent fit to the numerical data, and this enables determination of $\kappa(\omega, T)$. Our results for $\kappa$ and their scaling are shown in Fig. 4C,D of the main text at $\lambda = \lambda_c$. Corresponding results for $\lambda > \lambda_c$ appear in Figs. 6 and 7.

As for the dynamics spin susceptibility at the ordering wavevector, $\omega/T$ scaling works for $\lambda = \lambda_c$, but only for smaller values of $\omega/T$ for $\lambda > \lambda_c$. The plots of Fig. 7 are shown on a logarithmic frequency scale in Fig. 8 (as in Figs. 3 and 4D of the main text), which exposes the lower frequency range.

Finally, we complement plots of the resistivity induced by the disordered spin fluctuations in Fig. 4 of the main text at additional values of $\lambda$ in Fig. 9. The resistivity was computed from the numerical results for $\chi''_L(\omega)$, as specified in Ref. [2].

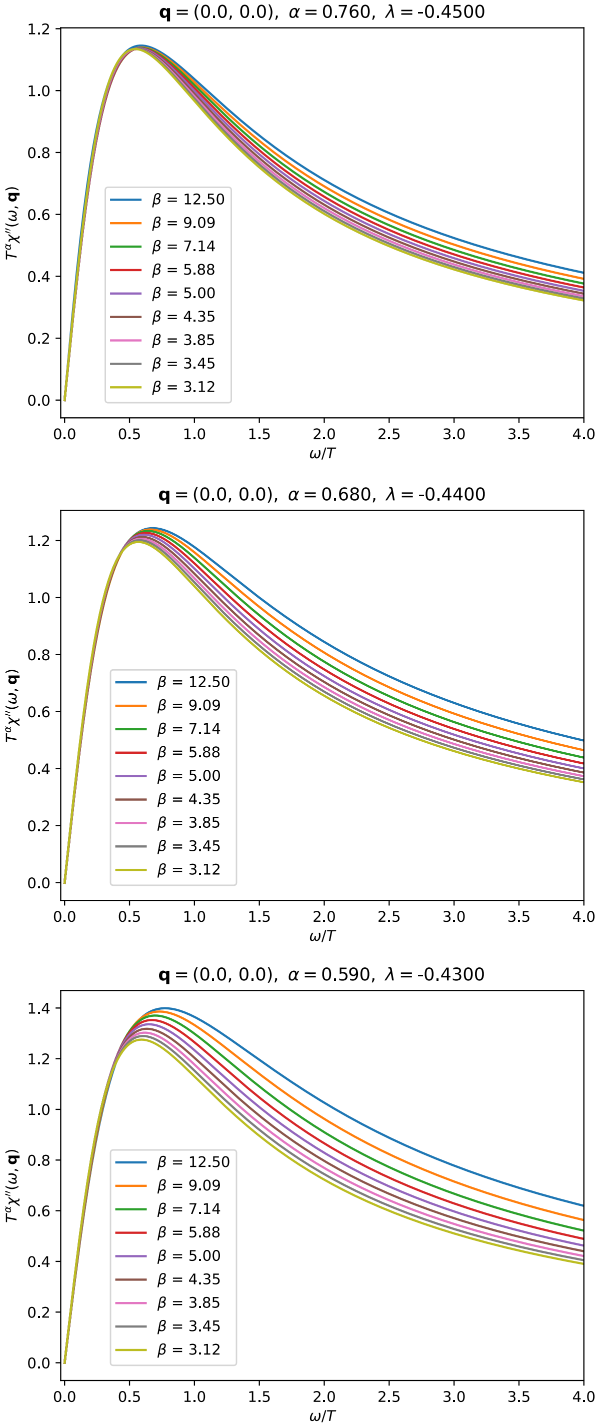


Supplementary Fig. 1. **Scaling plots of the dynamic spin susceptibility at the ordering wavevector for** $\lambda > \lambda_c = -0.4586$. Results for $\lambda = \lambda_c$ are in Fig. 4B in the main text.

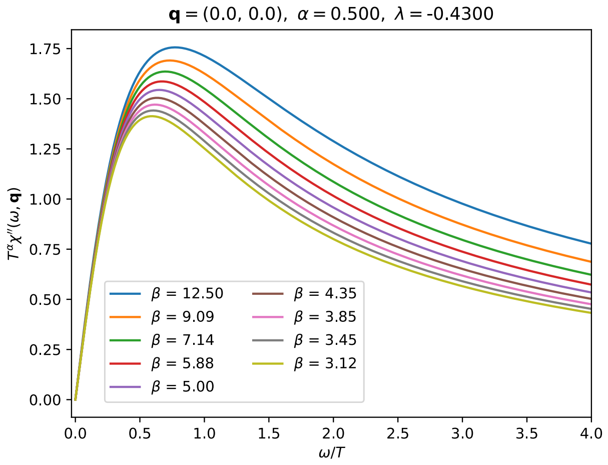


Supplementary Fig. 2. **Scaling plots of the dynamic spin susceptibility at the ordering wavevector for** $\lambda = -0.43$. A smaller value of the exponent $\alpha$ is used in comparison to the corresponding plot in Fig. 1.

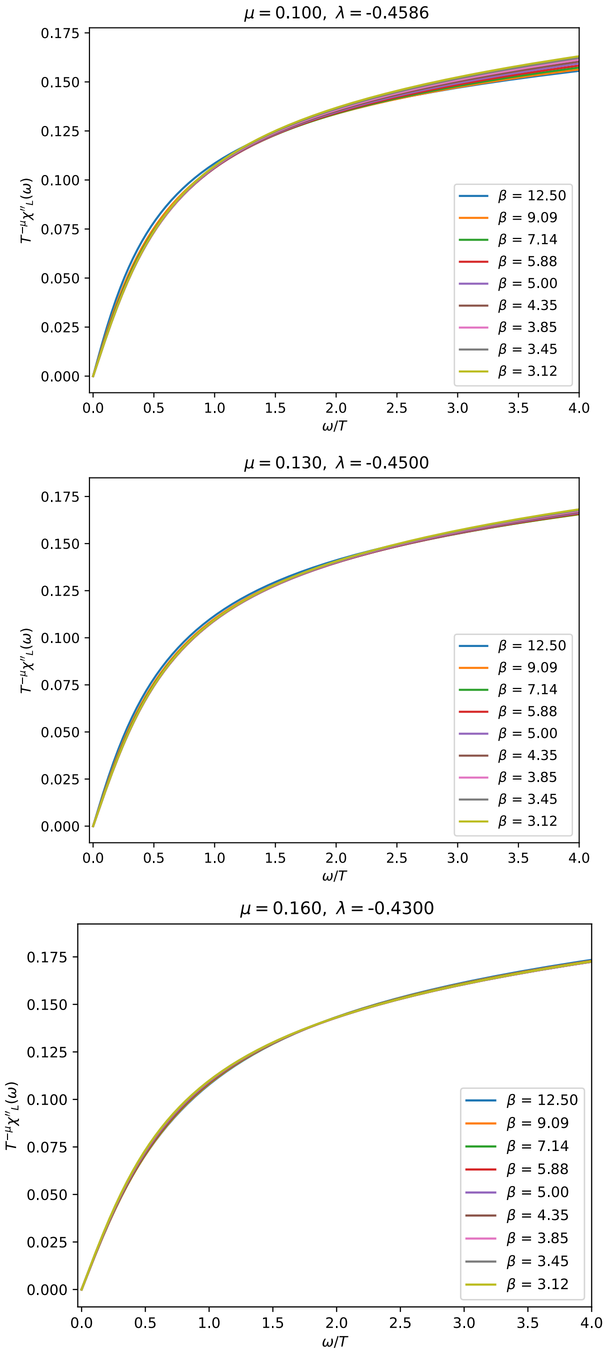


Supplementary Fig. 3. **Scaling plots of the local dynamic spin susceptibility for** $\lambda \geq \lambda_c = -0.4586$.

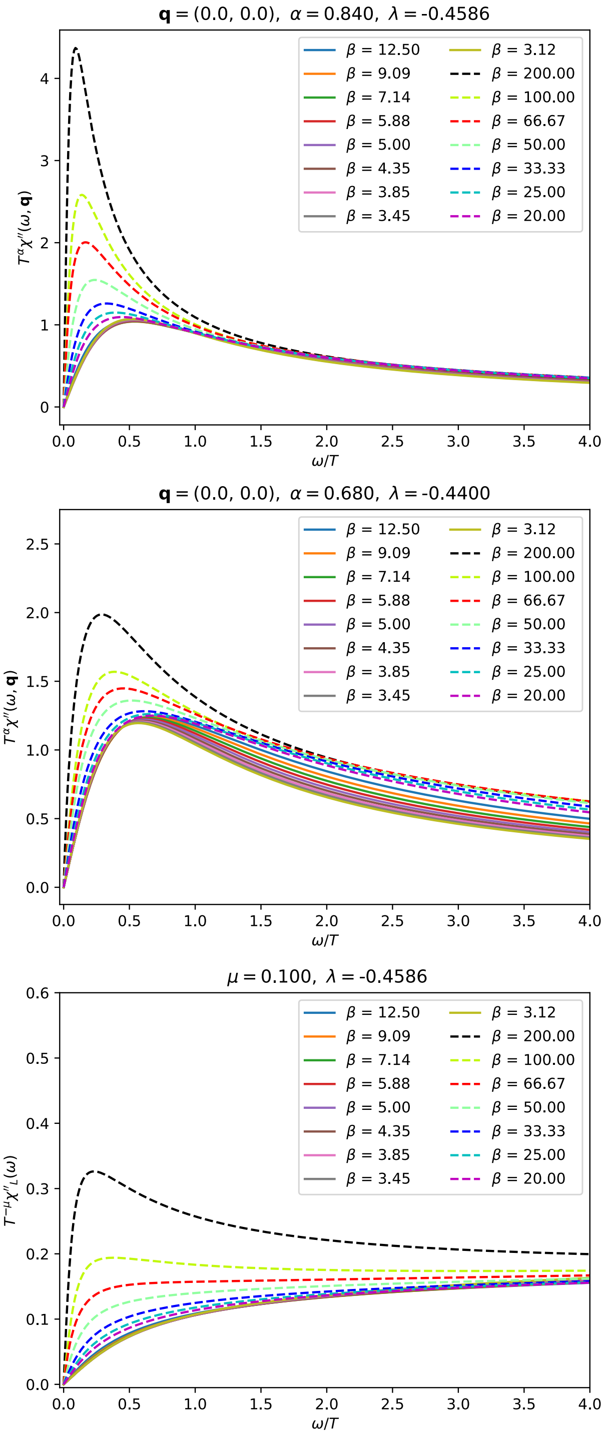


Supplementary Fig. 4. **Breakdown of scaling at very low temperatures**. The dynamic susceptibilities at lower temperatures. Compare to the plots in Fig. 4 of the main text and Fig. 3.

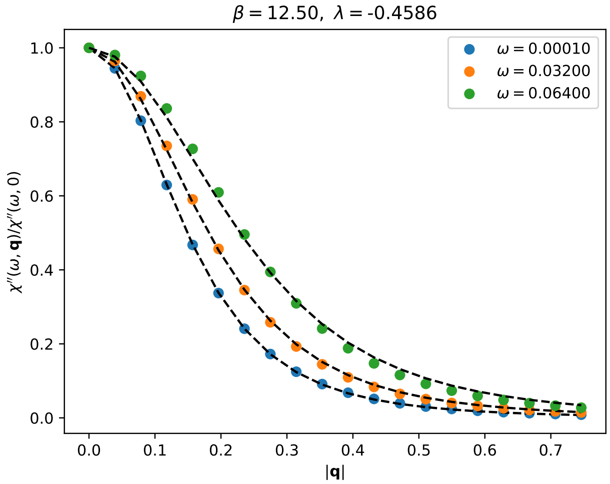


Supplementary Fig. 5. **Fits of the dynamic spin susceptibility to (S23), allowing determination of $\kappa(\omega, T)$.**

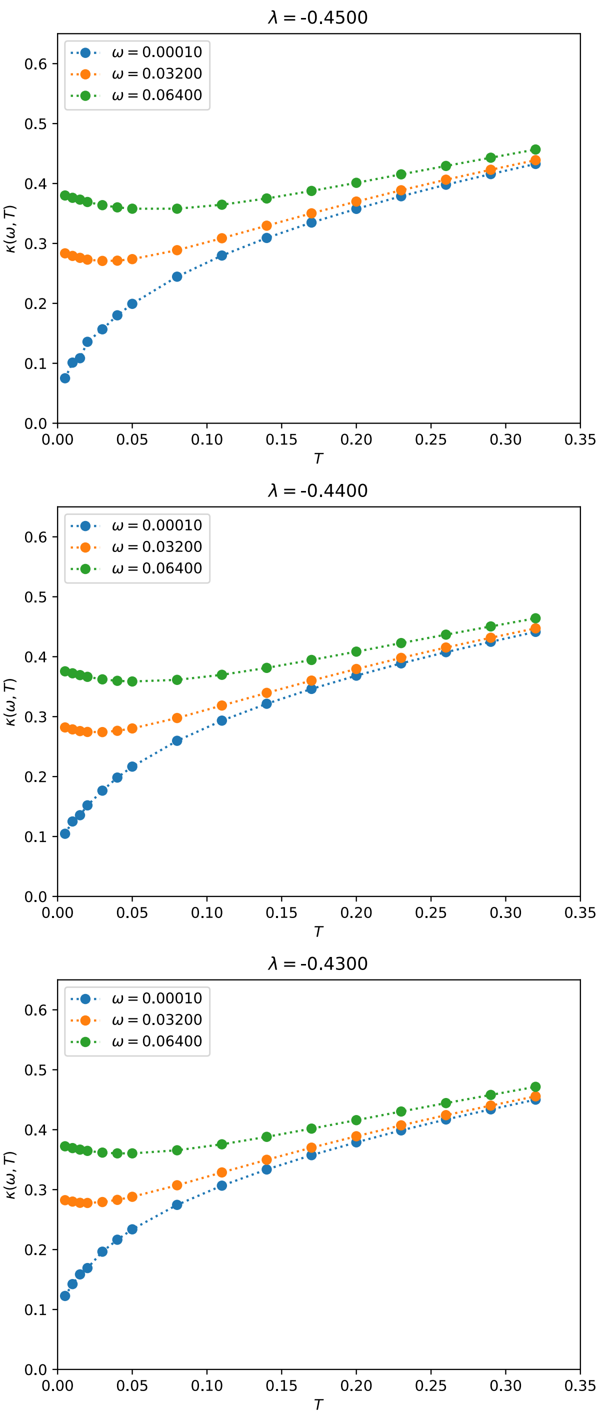


Supplementary Fig. 6. **Plots of $\kappa$ for $\lambda > \lambda_c = -0.4586$.** Results for $\lambda = \lambda_c$ are in Fig. 4C in the main text.

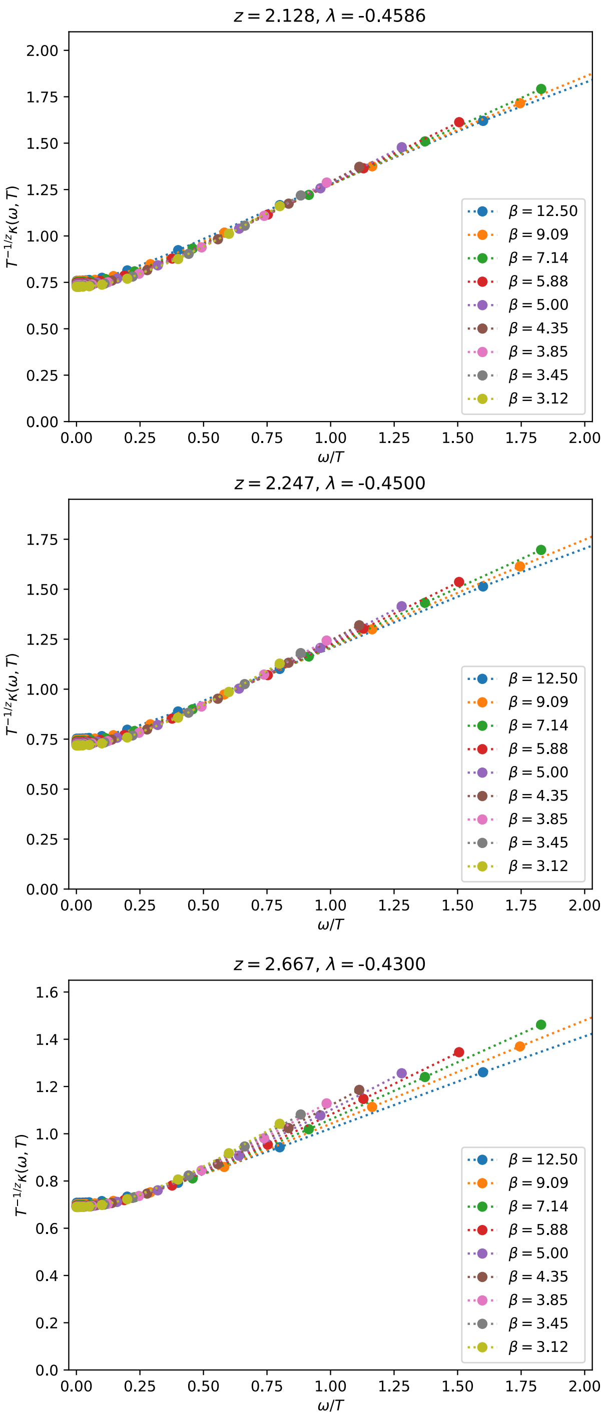


Supplementary Fig. 7. **Scaling plots of $\kappa$ for $\lambda \geq \lambda_c = -0.4586$.**

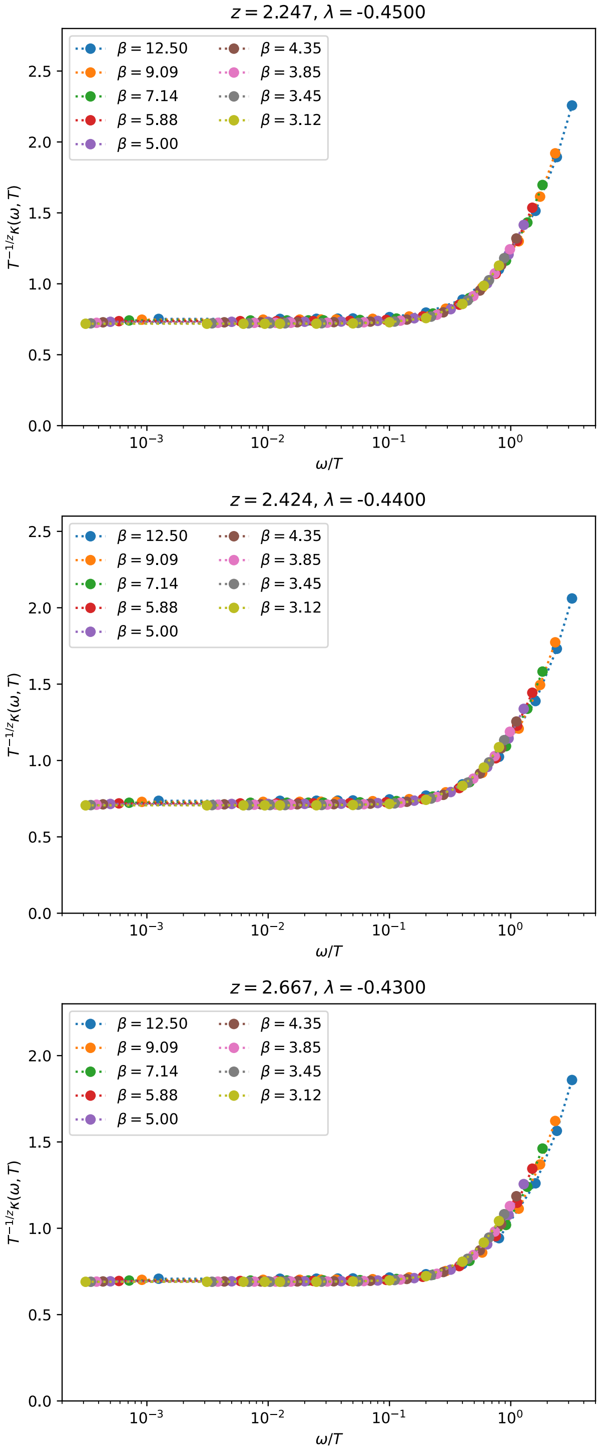


Supplementary Fig. 8. **Scaling plots of $\kappa$ for $\lambda > \lambda_c = -0.4586$**. As in Fig. 7, but with a logarithmic frequency axis. Compare to the experimental results in Figs. 3E and 4D of the main text.

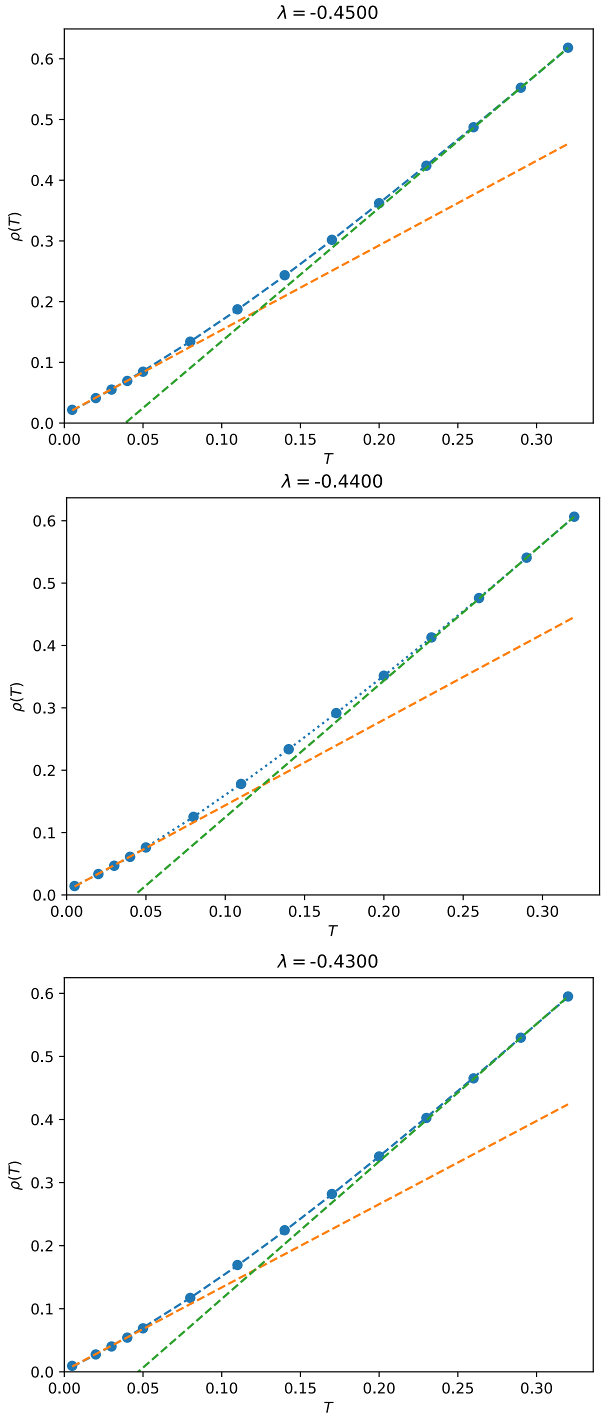


Supplementary Fig. 9. **Resistivity as a function of temperature**. As in Fig. 4F of the main text.

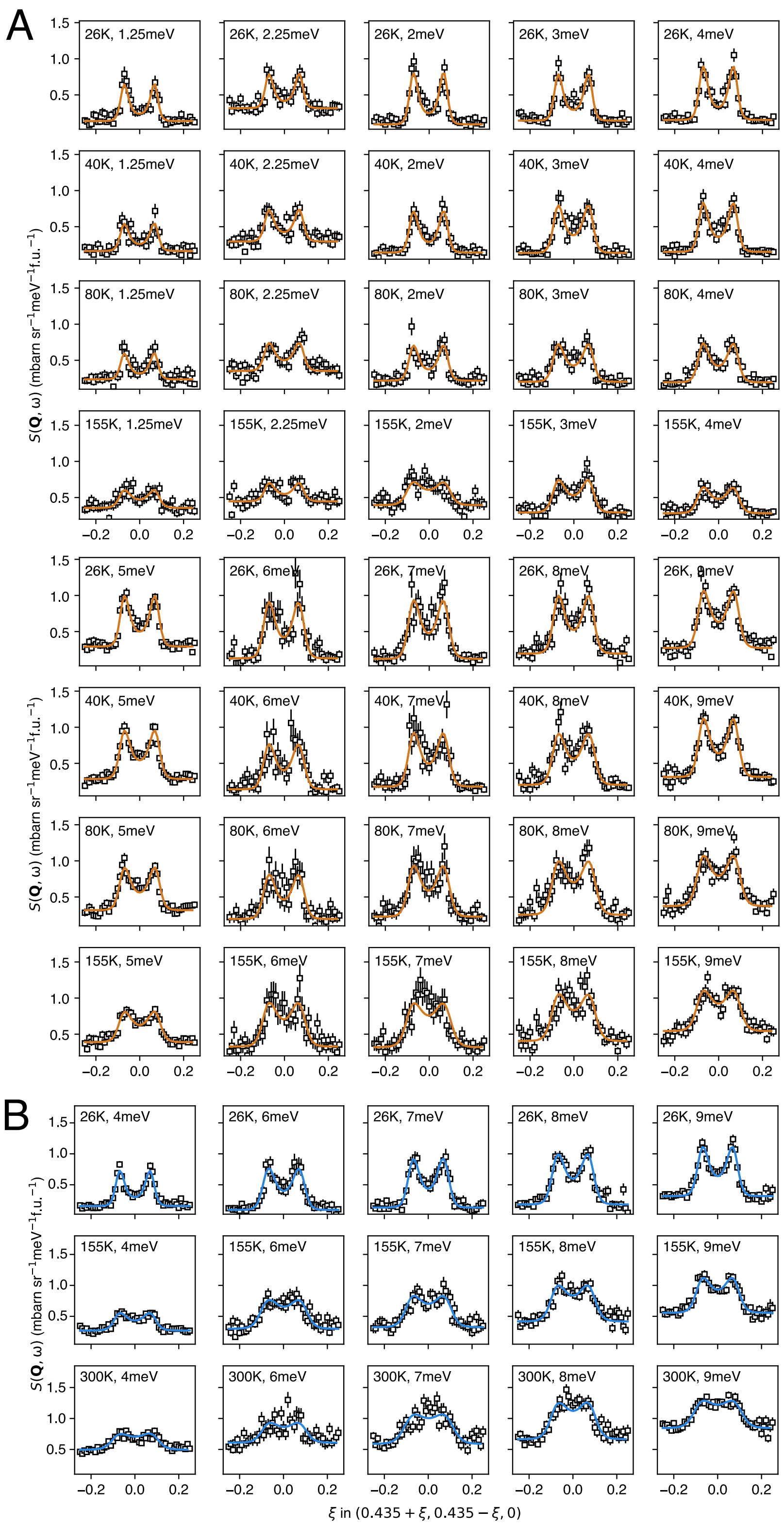


Supplementary Fig. 10. A representative selection of $S(\mathbf{Q}, \omega)$ cuts and fits to the model described in the main text. (**A**) Experiment 1. (**B**) Experiment 2.

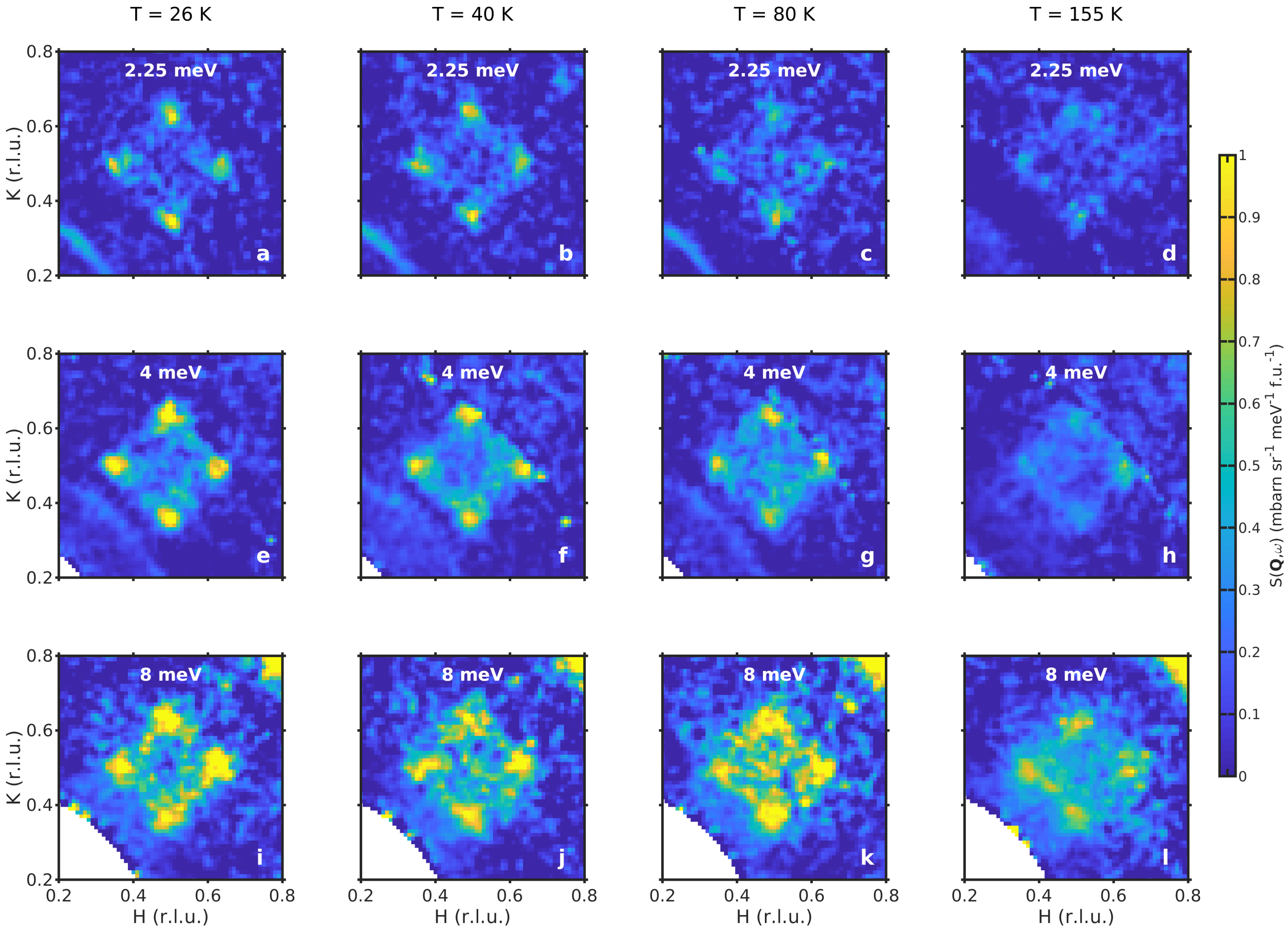


Supplementary Fig. 11. Slices of $S(\mathbf{Q},\omega)$ with subtraction of a flat background (see section 2-D **Q** Slices) for various energies and temperatures. Data at T=26K, 40K and 80K is from experiment 1 and T=155K from experiment 2.

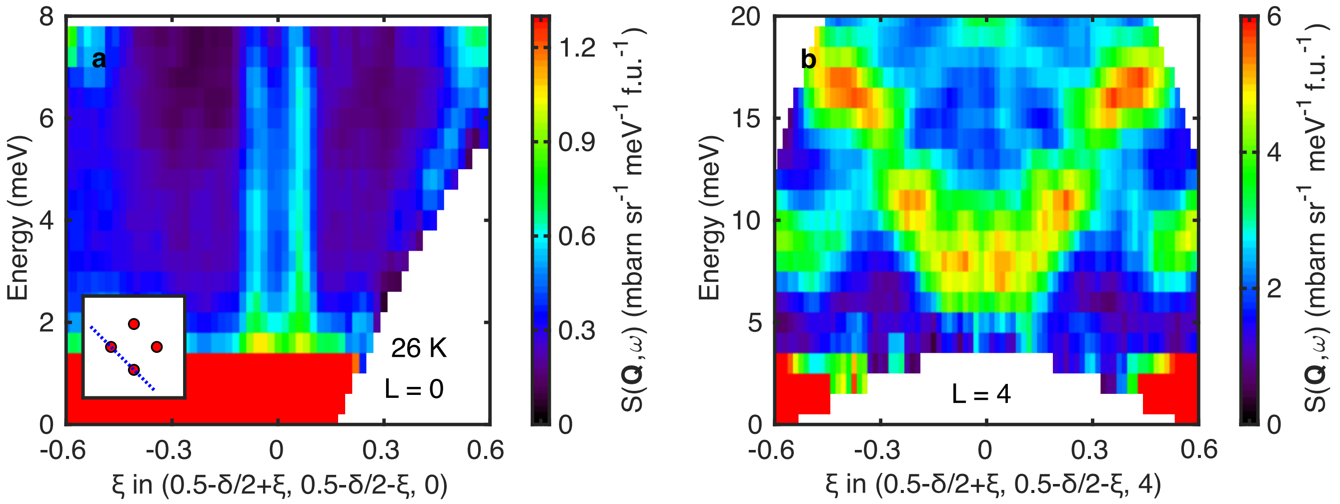


Supplementary Fig. 12. **Spin fluctuations and phonons in $La_{2-x}Sr_xCuO_4$ ($x = 0.22$) near $\mathbf{Q}_\delta$.** $S(\mathbf{Q}, \omega)$ as a function of energy and wavevector along a trajectory through two incommensurate wave vectors $\mathbf{Q}_\delta$ = (0.5-$\delta$, 0.5, $L$) and (0.5, 0.5-$\delta$, $L$) (see inset to panel **a**). Integration ranges are **a** $L \in [-1, 1]$ and **b** $L \in [3.8, 4.2]$. Strong phonons are observed (panel **b**) for $L \approx 4$, but these are not visible near $L = 0$ (panel **a**) where spin fluctuations are seen. Data were collected on LET (panel **a**) and MERLIN (panel **b**).

## SUPPLEMENTARY REFERENCES

[1] G. Aeppli, T. E. Mason, S. M. Hayden, H. A. Mook, and J. Kulda, Nearly singular magnetic fluctuations in the normal state of a high-$T_c$ cuprate superconductor, Science **278**, 1432 (1997).

[2] A. A. Patel, P. Lunts, and S. Sachdev, Localization of overdamped bosonic modes and transport in strange metals, Proc. Natl. Acad. Sci. **121**, e2402052121 (2024).

[3] S. Sachdev, The foot, the fan, and the cuprate phase diagram: Fermi-volume-changing quantum phase transitions, Physica C **633**, 1354707 (2025).

[4] J. A. Hertz, Quantum critical phenomena, Phys. Rev. B **14**, 1165 (1976).

[5] A. J. Millis, Effect of a nonzero temperature on quantum critical points in itinerant fermion systems, Phys. Rev. B **48**, 7183 (1993).

[6] I. Esterlis, H. Guo, A. A. Patel, and S. Sachdev, Large-$N$ theory of critical Fermi surfaces, Phys. Rev. B **103**, 235129 (2021).

[7] A. A. Patel, H. Guo, I. Esterlis, and S. Sachdev, Universal theory of strange metals from spatially random interactions, Science **381**, 790 (2023).

[8] P. A. Lee and T. V. Ramakrishnan, Disordered electronic systems, Rev. Mod. Phys. **57**, 287 (1985).

[9] A. A. Patel, P. Lunts, and M. S. Albergo, Strange metals and planckian transport in a gapless phase from spatially random interactions, Phys. Rev. X **15**, 031064 (2025).

[10] C. Li, D. Valentinis, A. A. Patel, H. Guo, J. Schmalian, S. Sachdev, and I. Esterlis, Strange Metal and Superconductor in the Two-Dimensional Yukawa-Sachdev-Ye-Kitaev Model, Phys. Rev. Lett. **133**, 186502 (2024).

[11] J. H. Van Vleck, Note on the interactions between the spins of magnetic ions or nuclei in metals, Rev. Mod. Phys. **34**, 681 (1962).

[12] J. A. Hoyos, C. Kotabage, and T. Vojta, Effects of Dissipation on a Quantum Critical Point with Disorder, Phys. Rev. Lett. **99**, 230601 (2007).

[13] T. Vojta, C. Kotabage, and J. A. Hoyos, Infinite-randomness quantum critical points induced by dissipation, Phys. Rev. B **79**, 024401 (2009).

[14] T. Vojta, Phases and phase transitions in disordered quantum systems, in *Lectures on the Physics of Strongly Correlated Systems XVII: Seventeenth Training Course in the Physics of Strongly Correlated Systems*, American Institute of Physics Conference Series, Vol. 1550, edited by A. Avella and F. Mancini (AIP, 2013) pp. 188–247, arXiv:1301.7746.

[15] A. Del Maestro, B. Rosenow, M. Müller, and S. Sachdev, Infinite Randomness Fixed Point of the Superconductor-Metal Quantum Phase Transition, Phys. Rev. Lett. **101**, 035701 (2008).

[16] S. M. Hayden, G. Aeppli, R. Osborn, A. D. Taylor, T. G. Perring, S. W. Cheong, and Z. Fisk, High-energy spin-waves in $La_2CuO_4$, Phys. Rev. Lett. **67**, 3622 (1991).
